# Natural Form of Noncytolytic Flexible Human Fc as a Long-Acting Carrier of Agonistic Ligand, Erythropoietin

**DOI:** 10.1371/journal.pone.0024574

**Published:** 2011-09-16

**Authors:** Se Jin Im, Sang In Yang, Se Hwan Yang, Dong Hoon Choi, So Young Choi, Hea Sook Kim, Do Soo Jang, Kyeong Sik Jin, Yo-Kyung Chung, Seung-Hee Kim, Sang Hoon Paik, Yoo Chang Park, Moon Koo Chung, Yong Bum Kim, Kang-Hyun Han, Kwan Yong Choi, Young Chul Sung

**Affiliations:** 1 Division of Molecular and Life Sciences, POSTECH, Pohang, Republic of Korea; 2 Research Institute, Genexine Co., Seongnam, Republic of Korea; 3 Pohang Accelerator Laboratory, POSTECH, Pohang, Republic of Korea; 4 Central Research Institute, Green Cross Co., Yongin, Republic of Korea; 5 Korea Institute of Toxicology, Korea Research Institute of Chemical Technology, Daejon, Republic of Korea; Institut national de la santé et de la recherche médicale (INSERM), France

## Abstract

Human IgG1 Fc has been widely used as a bioconjugate, but exhibits shortcomings, such as antibody- and complement-mediated cytotoxicity as well as decreased bioactivity, when applied to agonistic proteins. Here, we constructed a nonimmunogenic, noncytolytic and flexible hybrid Fc (hyFc) consisting of IgD and IgG4, and tested its function using erythropoietin (EPO) conjugate, EPO-hyFc. Despite low amino acid homology (20.5%) between IgD Fc and IgG4 Fc, EPO-hyFc retained “Y-shaped” structure and repeated intravenous administrations of EPO-hyFc into monkeys did not generate EPO-hyFc-specific antibody responses. Furthermore, EPO-hyFc could not bind to FcγR I and C1q in contrast to EPO-IgG1 Fc. In addition, EPO-hyFc exhibited better in vitro bioactivity and in vivo bioactivity in rats than EPO-IgG1 Fc, presumably due to the high flexibility of IgD. Moreover, the mean serum half-life of EPO-hyFc(H), a high sialic acid content form of EPO-hyFc, was approximately 2-fold longer than that of the heavily glycosylated EPO, darbepoetin alfa, in rats. More importantly, subcutaneous injection of EPO-hyFc(H) not only induced a significantly greater elevation of serum hemoglobin levels than darbepoetin alfa in both normal rats and cisplatin-induced anemic rats, but also displayed a delayed time to maximal serum level and twice final area-under-the-curve (AUC_last_). Taken together, hyFc might be a more attractive Fc conjugate for agonistic proteins/peptides than IgG1 Fc due to its capability to elongate their half-lives without inducing host effector functions and hindering bioactivity of fused molecules. Additionally, a head-to-head comparison demonstrated that hyFc-fusion strategy more effectively improved the in vivo bioactivity of EPO than the hyperglycosylation approach.

## Introduction

The application of Fc-fusion strategies to therapeutic proteins has become increasingly popular in recent years, as evidenced by the appearance of commercial products such as Orencia (CTLA-4-Fc), Amevive (LFA3-Fc) and Enbrel (TNFR-Fc) [1]. Fusion with an Fc fragment could extend the serum half-life of conjugated therapeutics for two reasons: it is recycled via the neonatal Fc receptor (FcRn) and creates a larger effective molecular size [2]. After internalization by fluid-phase pinocytosis, Fc-fusion proteins bind to FcRn under the acidic pH conditions of endosomes and are released at the basic pH level of blood, a pathway known to be the main mechanism responsible for the long serum half-life of IgG [3]. In addition, the larger hydrodynamic size of Fc-fusion proteins inhibits their translocation from blood to extravascular tissues and reduces their renal clearance [4].

Human IgG1 has been one of the most widely used human immunoglobulin (Ig) Fc molecules, but it is inefficient in generating long-acting agonistic proteins. This is largely because human IgG1 is able to bind to the Fcγ receptor I (FcγR I) or complement component 1q (C1q), resulting in antibody-dependent cellular cytotoxicity (ADCC) or complement-dependent cytotoxicity (CDC) of target cells *in vitro* and *in vivo* [5]. Directed mutation or deletion of FcγR I or C1q binding sites has been employed in an attempt to disrupt this binding and eliminate cytotoxicity [6]. However, because these mutant residues must be exposed on the protein exterior in order to disrupt binding to counterpart surfaces on the cognate receptor, such mutagenesis strategies have led to concerns that the mutated or deleted sequences might cause undesirable immune responses. An alternative approach is to use IgG4 Fc, which cannot bind FcγR III or C1q [7]. However, IgG4 Fc still retains moderate binding affinity for FcγR I and its hinge region is less flexible than that of IgG1 Fc. More problematically, it has been reported that IgG4 Fc can form two intrachain disulfide bonds, which can cause the generation of monovalent half-molecules [8]. Another strategy for circumventing the weaknesses of IgG1 is to construct hybrid Fc molecules. On such hybrid molecules, IgG1/IgG2 cannot bind to FcγR I but is still able to bind to C1q [9]. Exploiting the fact that IgG2 does not bind to FcγR I/III and IgG4 does not activate complement, two groups independently constructed and tested IgG2/IgG4 hybrids. An et al. [10] constructed a hybrid IgG2/IgG4, substituting residues of the IgG2 backbone that are important for C1q binding with the corresponding IgG4 residues. The IgG2 amino acids flanking these substitutions are identical to those in the native IgG4 sequence; thus, the stretches of amino acids with substitutions in IgG2 are also present in IgG4. Another IgG2/IgG4 hybrid, eculizumab (Soliris), is a monoclonal antibody against the terminal complement protein C5 consisting of the hinge and CH1 regions of IgG2 and the CH2 and CH3 regions of IgG4 [11]. In this hybrid, IgG2 and IgG4 were joined at a restriction endonuclease site-containing oligonucleotide with flanking sequences identical to those of IgG2 and IgG4. However, without mutating or substituting sites, it is difficult to completely avoid ADCC and CDC. In addition, this strategy imposes more severe steric hindrance between neighboring molecules and diminishes bioactivity due to the relatively low hinge flexibility of IgG2 (32°).

Erythropoietin (EPO) is a naturally occurring, 30-kDa (165 amino acid) hematopoietic growth factor produced by the kidney [12]. As the primary regulator of erythropoiesis, EPO stimulates the proliferation of bone marrow erythroid precursor cells and promotes their differentiation into red blood cells (RBCs) in response to a decrease in tissue oxygenation [13]. However, the terminal serum half-life (t_1/2_) of recombinant EPO (r-EPO) ranges from 5 to 11 hours (hrs) after intravenous (IV) administration, necessitating frequent administration [14]. It has been reported that the increase in the RBC population in response to administration of r-EPO is mainly regulated by the persistence of EPO exposure [15]. Thus, enhancing the duration of serum EPO could substantially increase the dosing interval, potentially providing an important therapeutic benefit. Two different strategies—hyperglycosylation [16] and polyethylene glycol (PEG)-conjugation [17]—have been developed to extend the serum half-life of EPO. One well-established modified EPO, darbepoetin alfa (Aranesp, NESP), is a heavily glycosylated EPO analogue that has been used for 10 years to treat anemia. Unlike native EPO, which contains one O-linked and three N-linked carbohydrate chains with a maximum of 14 sialic acids [18], darbepoetin alfa was engineered to include two additional N-linked carbohydrate chains containing a maximum of 22 sialic acids [16]. Micera (C.E.R.A), another recently introduced and commercially available modified EPO, is a pegylated form made by linking the N-terminal amino lysine group of epoetin beta with methoxy polyethylene glycol-succinimidyl butanoic acid through amide bonds [17]. Both of these modified EPOs exhibit reduced ability to bind the EPO receptor (EPOR) compared to r-EPO; however, they exhibit greater *in vivo* biological activity as a result of their longer serum half-lives [19], [20]. These properties permit less frequent dosing of patients while maintaining biological effects.

In this study, we developed a novel type of hybrid Fc (hyFc), which is nonimmunogenic and noncytolytic Fc having on intact Ig structure, consisting of human IgD and IgG4 without any additional artificial amino acids. In addition, EPO fused with hyFc (EPO-hyFc) achieved better *in vitro* and *in vivo* bioactivity than EPO-IgG1 Fc, possibly owing to the flexible hinge region. More significantly, a head-to-head comparison with darbepoetin alfa, which has been extensively used in clinic, showed that the hyFc-fusion strategy more effectively improved recovery from cisplatin-induced anemia as well as the *in vivo* bioactivity of EPO in normal rats than did the hyperglycosylation approach, especially after subcutaneous (SC) administration. Our findings suggest that hyFc could be easily used for manufacturing a second generation of other therapeutic proteins commercially available now, such as G-CSF, human growth hormone and factor VII. More importantly, hyFc could be applied to therapeutic short peptide such as GLP-1 (30 aa, t_1/2_ 2 min [21]) and BNP (32 aa, t_1/2_ 20 min [22]), which have not been used in clinics due to very short half-lives *in vivo*.

## Materials and Methods

### Animals

Sprague-Dawley (SD) male rats at 6 to 8 weeks old were purchased from Japan SLC. Rats are housed under specific pathogen free (SPF) conditions in an approved animal facility at Postech Biotech Center. Rat experiments were performed in accordance with the procedures outlined in the guide for the care and use of laboratory animals and approved by POSTECH animal committee (2010-02-0003). Male and Female cynomolgus monkeys at 2 to 3 years old were purchased from Guangxi Grandforest scientific primate company (China) and were acclimated for a 3-month period in Korea Institute of Toxicology. Monkeys were also cared for in accordance with the guidelines outlined in the guide for the care and use of laboratory animals [23].

### Construction of cell lines

**Figure 1:**
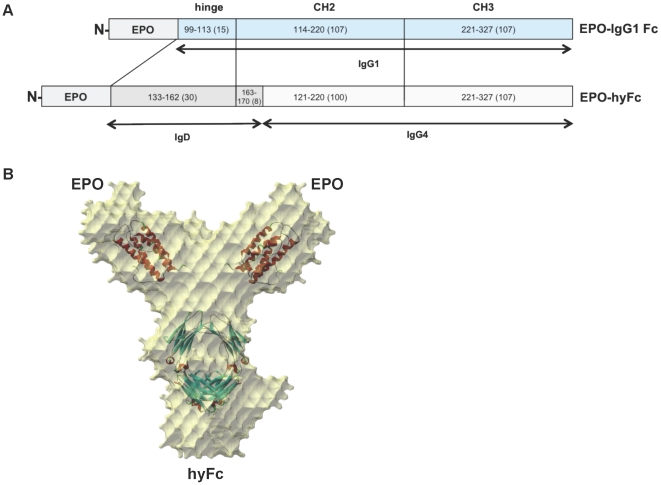
Schematic diagram of EPO-IgG1 Fc and EPO-hyFc, and X-ray structure of EPO-hyFc. (A) Schematic diagram of hyFc and IgG1 Fc fused with EPO. The numbers in the boxes denote the amino acid numbers from the CH1 region of each immunoglobulin. (B) The structure of EPO-hyFc, determined by small-angle X-ray scattering, was reconstituted from the known structure of EPO. The sheets in the structure were fit based on the crystal structure.

The coding sequences for human EPO, the human IgG1 Fc (hinge, CH2 and CH3 domains) that contains the same amino acid sequences of Fc used for CTLA4Ig, and the hybrid Fc that contains the 30 amino acids (aa) (133^th^–162^nd^) of the C-terminal IgD hinge, the 8 aa (SHTQPLGV; 163^rd^–170^th^) of the N-terminal IgD CH2 domain, the 100 aa (121^st^–220^th^) of the IgG4 CH2 domain, and the 107 aa (221^st^–327^th^) of the IgG4 CH3 domain were obtained from codon-optimization synthesis (TOP Gene Technologies, Montreal, Canada) (Fig. 1A). These were moved into the pAD11 vector showing a 12-fold increase in expression level compared with the RcCMV vector (Invitrogen, CA, USA) (unpublished data). CHO/DHFR^−/−^ cells (Chinese hamster ovary cells, DG44, kindly provided by Dr. Chain of Columbia University) were transfected with genes for EPO-IgG1 Fc and EPO-hyFc according to the conventional CaPO_4_ coprecipitation methods [24]. Transfected cells were selected for growth in the presence of 0.18 µM methothrexate (Sigma-Aldrich, MO, USA) and adapted in suspension culture using Ex-Cell CHO DHFR^−^ animal-component free medium (SAFC, MO, USA).

### Purification of EPO-hyFc and EPO-IgG1 Fc

For purification, HiTrap recombinant protein A FF columns (Amersham Biosciences, NJ, USA) were equilibrated with phosphate-buffered saline (PBS) (pH 7.0). The filtered supernatants were added to the columns and eluted with 0.1 M sodium citrate (pH 3.0) followed by neutralization with 1 M Tris-HCl (pH 9.0). Eluted samples were passed through an additional gel filtration column (Superdex 200 and Acta Explorer; GE Healthcare, NJ, USA) with 100 nM sodium citrate buffer (pH 4.0). Less glycosylated EPO-hyFc (EPO-hyFc(L)) was obtained from the flow-through after loading onto a Q sephrose FF column (GE Healthcare, NJ, USA); highly glycosylated EPO-hyFc (EPO-hyFc(H)) was also eluted with 10 mM sodium phosphate, 100 mM Arginine, 50 mM NaCl (pH 6.9). The proteins were finally obtained after undergoing dialysis with a membrane (MWCO 12–14K; Spectrum, CA, USA) more than three times.

### Small X-ray scattering (SAXS)

SAXS experiments were performed at the beamline 4C1 of the Pohang Light Source as previously described [25]. The low resolution structure of EPO-hyFc was reconstituted by using an ab initio shape-determination program, GASBOR [26]. The structural models were rendered using Discovery Studio 1.6 (Accelrys Inc.) on the basis of two structures of EPO (1BUY) and IgG (3DNK).

### FcγR I- and C1q-binding assays

The FcγR I- and C1q-binding activity of EPO-hyFc was determined by FcγR I and C1q ELISA as described [10] with minor modification. EPO-hyFc, EPO-IgG1 Fc, Herceptin (Roche, Basel, Switzerland) and Enbrel (Amgen, CA, USA) with 2-fold or 3-fold serial dilution (starting concentration: 2 µg/ml for FcγR I or 100 µg/ml for C1q) in PBS were coated in 96-well immunoplates (Nunc, Copenhagen, Denmark) and left overnight at 4°C. After blocking, 100 µl of 2 µg/ml human FcγR I (R&D, MN, USA) or human C1q (AbD SEROTEC, NC, USA) was added. Next, sequential treatment of 2 µg/ml biotin-conjugated anti-human IgG1 antibody (R&D, MN, USA) and 1∶3000-diluted streptavidin-HRP (BD Bioscience, CA, USA) or treatment of 1∶400-diluted HRP-conjugated anti-C1q antibody (AbD SEROTEC, NC, USA) was performed. After washing, 50 µl TMB substrate (KPL, MD, USA) was added; the reaction was stopped by the addition of 50 µl 2 N H_2_SO_4_. The optical density (OD) was determined at 450 nm after subtracting the OD at 590 nm.

### In vitro bioassay of EPO derivatives

*In vitro* bioassay of EPO derivatives was carried out as described [27] with minor modification. The human leukemic F36E cells (RIKEN BRC, Japan) were maintained with 5 IU/ml darbepoetin alfa (Aranesp; Amgen, CA, USA) in assay media. After 1 day of darbepoetin alfa starvation, starved F36E cells were resuspended at 1×10^5^ cells/ml and a total of 100 µl (1×10^4^ cells) of the cell suspension was aliquoted to each test well. Serial 2-fold dilutions of r-EPO (batch 3; EDQM, Strasbourg, France), darbepoetin alfa, EPO-hyFc and EPO-IgG1 Fc were also prepared in assay media. A total of 100 µl of the diluted EPO derivatives were added to the triplicate wells. After 3 days, 20 µl of CellTiter 96 Aqueous One Solution (Promega, WI, USA) was added to each well and the plates were incubated at 37°C in the tissue culture incubator for 4 hrs. Absorbance of the wells was read at 490 nm using a microplate reader.

### Colony assays

Methocult media M3234 (Stem Cell Technologies, BC, USA) was used for colony assays according to the manufacturer's instructions with minor modification. Bone marrow cells isolated from 6-week-old C57bl/6 mice were suspended in Iscove's Modified Dulbecco's Media (IMDM) containing 2% fetal bovine serum (FBS) and loaded onto 35 mm non-adhesive culture dishes (1×10^5^ cells/ml/dish; Stem Cell Technologies, BC, USA) with 1 nM of r-EPO, darbepoetin alfa, EPO-hyFc, or EPO-IgG1 Fc. All samples were cultured in a humidified incubator at 37°C with 5% CO_2_. The colonies were counted directly using an inverted microscope on day 2 for erythroid colony-forming unit colonies (CFU-E) and on day 8 for erythroid burst-forming unit colonies (BFU-E).

### Pharmacokinetic study in rats

To compare the half-life of EPO-hyFc and EPO-IgG1 Fc, six SD male rats were treated with a single IV injection of 400-pmol/kg r-EPO, EPO-hyFc and EPO-IgG1 Fc. After IV injection, blood samples were drawn before injection and at timepoints up to 168 hpi (hours post injection).

To examine comparative pharmacokinetic profiles of darbepoetin alfa and EPO-hyFc(H), groups of ten SD male rats were injected intravenously or subcutaneously with 800-pmol/kg darbepoetin alfa and EPO-hyFc(H). After IV and SC administration, blood samples were drawn from 5 rats each at alternative time points before injection and at timepoints up to 168 hpi. Sera from each sample were stored in a −80°C freezer. All samples obtained at each time point were tested for the quantification of EPO by EPO ELISA kit (R&D, MN, USA) according to the manufacturer's protocol.

### Pharmacodynamic study in rats

After a single IV administration of 400-pmol/kg r-EPO, EPO-IgG1 Fc, EPO-hyFc and buffer into six male SD rats, blood samples were collected daily up to 8 days post injection (dpi) in a K3 EDTA vacutainer tube (BD Bioscience, CA, USA) to inhibit coagulation. One milliliter of Retic-COUNT™ solution (thialzole orange; BD Bioscience, CA, USA) was aliquoted into polystyrene tubes (12×75 mm) and 5 µl of each blood sample was added. After incubation at room temperature for 30 min under dark conditions, the number of reticulocytes in these samples was measured by FACS Calibur (BD Bioscience, CA, USA).

A dose of 135-pmol/kg darbepoetin alfa and EPO-hyFc(H) as well as buffer was administrated into five SD male rats via IV and SC routes. For establishing the chemical-induced anemic models, 6 mg/kg of cisplatin (Joongwea Pharmaceuticals, Korea) was treated via intraperitoneal route into SD male rats. Nine days after cisplatin treatment, 100-pmol/kg darbepoetin alfa and EPO-hyFc(H) were administered via IV (n = 8) and SC (n = 6) routes. Blood samples were drawn before injection and at timepoints up to 27 dpi and collected in a K3 EDTA vacutainer tube. Hemoglobin (Hb) level was determined using an automated CBC counter (MELET SCHLOESING Lab., France).

### Immunological and hematological responses by EPO-hyFc(H) treatment in monkeys

Cynomolgus male and female monkeys were treated with 1, 3, 10 µg/kg of EPO-hyFc(H) or vehicle control twice a week for four weeks (9 times; from day 1 to 29) via IV route (n = 10, buffer control and 10 µg/kg-treated groups; n = 6, 1 and 3 µg/kg-treated groups). Anti-EPO-hyFc specific antibody responses were determined at day 15, 29 (n = 6 or 10 in all groups), 44, 58 and 72 (n = 4 in vehicle control or 10 µg/kg-treated group) by ELISA as described [28], [29]. EPO-hyFc(H) was diluted to 1 µg/ml in PBS and coated in 96-well immunoplates (Nunc, Copenhagen, Denmark). After blocking, 100 µl of monkey sera or chicken polyclonal anti-EPO antibody (Abcam, SF, USA) as a positive control was added, followed by sequential treatment of 100 µl of biotin-conjugated secondary antibody and 1∶3000-diluted streptavidin-HRP (BD Bioscience, CA, USA). After washing, 100 µl TMB substrate (KPL, MD, USA) was added and the reaction was stopped by the addition of 100 µl 2 N H_2_SO_4_. Normalized negative cut off was determined by using monkey blank sera and monkey sample sera showing higher optical density at 450 nm than the negative cut off were re-tested by an immunodepletion test, which examines the difference in the OD between before and after reacting monkey sera with 1 µg/ml of EPO-hyFc(H). When the difference in the OD was more than 30%, the sample was regarded as positive. Before the administration (day 0) and one day after the last administration (day 30), Hb concentration, RBC counts and hematocrits were determined by ADVIA120 Hematology System (Bayer, NY, USA).

### Data and Statistical analysis

PK parameters were determined using a non-compartmental approach using WinNonlin (Pharsight Corp., CA, USA) including terminal half-life (t_1/2_), area under the curve-last (AUC_last_, from time zero to the time of the last quantifiable concentration), maximum serum concentration (C_max_), initial serum concentration (C_0_) and time to C_max_ (T_max_). All values are expressed as the mean ± the standard error of the mean (SEM). A two-tailed student's t-test was used to examine statistical differences between the experimental groups.

## Results

### Characteristics of recombinant EPO-hyFc

Recombinant EPO-hyFc and EPO-IgG1 Fc (Fig. 1A) were produced from CHO cell lines and purified (>99% pure) from serum-free culture media by protein affinity chromatography and size exclusion chromatography as described in “Material and methods”. As expected, the purified EPO-hyFc protein consisted of a homodimer of approximately 130 kDa, which is slightly heavier than the EPO-IgG1 Fc protein (approximately 110 kDa) (Fig. S1); both proteins had similar pI values, indicating comparable carbohydrate content (data not shown). In contrast to the greater than 95% homology observed among other IgG subclasses, the amino acid homology between IgD Fc and IgG4 Fc is only 20.5%. Despite this low sequence homology, the “Y-shaped” structure of hyFc was confirmed by small X-ray scattering, indicating that the hyFc consisting of IgD Fc and IgG4 Fc retained an intact Ig-like structure (Fig. 1B).

**Figure 2:**
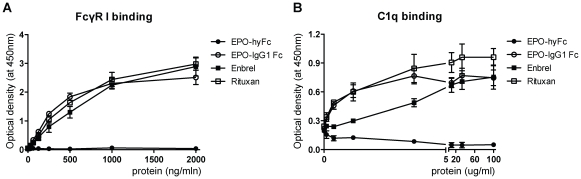
Binding capability of EPO-hyFc to FcγR I and C1q. Binding of EPO-hyFc (•), EPO-IgG1 Fc (○), Enbrel (▮), and Rituxan (□) to human FcγR I (A) and human C1q (B) was assessed by an indirect ELISA method. Data, presented as means ± SEMs, are obtained from three independent experiments.

Because hyFc contains the upper CH2 domain of IgD and the last CH2 and CH3 domains of IgG4, which do not have FcγR- or C1q-binding sites, we examined the binding affinity of EPO-hyFc to recombinant FcγR I and C1q proteins. Using ELISA, we found that EPO-hyFc was unable to bind either protein over a concentration range of 31.2 to 2000 ng/ml for FcγR I and 0.1 to 100 ug/ml for C1q (Fig. 2). In contrast, EPO-IgG1 Fc, Herceptin, and Enbrel used as positive controls bound both FcγR I and C1q. Additionally, when monkeys received 10 µg/kg of EPO-hyFc(H) 9 times via IV routes, there was mild and moderate bone marrow hyperplasia, which might be associated with erythropoiesis. However, no destructive feature was found in bone marrow where in vivo erythropoiesis occurs (Fig. S2), suggesting that hyFc would not induce antibody-mediated cytotoxicity.

### Improved bioactivity of EPO-hyFc over EPO-IgG1 Fc

**Figure 3:**
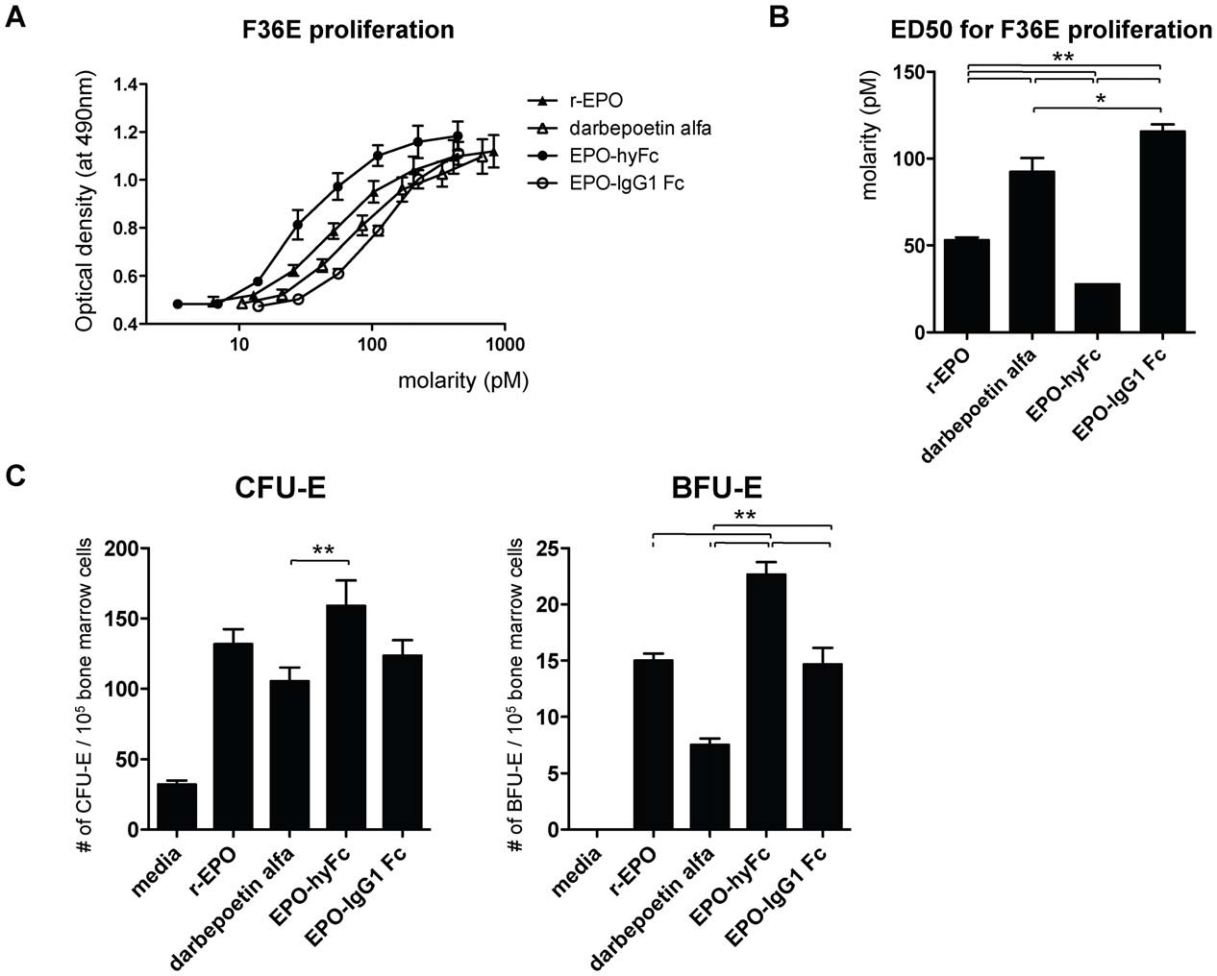
In vitro bioactivity of r-EPO, darbepoetin alfa, EPO-hyFc and EPO-IgG1 Fc. In vitro bioactivity of r-EPO (▴), darbepoetin alfa (△), EPO-hyFc (•), and EPO-IgG1 Fc (○) was determined by their effect on F36E cell proliferation shown as concentration dependence (in molar terms) (A) and ED_50_ (B) and on the number of CFU-Es and BFU-Es generated from bone marrow progenitor cells of C57bl/6 mice (C). Data, presented as means ± SEMs, are derived from four (F36E proliferation) and three (colony assays) independent experiments. (*p<0.05, **p<0.01)

To compare the *in vitro* bioactivity of EPO-hyFc and EPO-IgG1 Fc, we first evaluated the proliferation of EPO-responsive human leukemic F36E cells after 3 days of coculture with the two Fc-fused EPO proteins. r-EPO and darbepoetin alfa were used as controls. As expected, all EPO forms induced a concentration-dependent increase in cell number, as evidenced by the shape of the proliferation curve (Fig. 3A). The half-maximal effective dose of EPO-hyFc (ED_50_, 27.6 pM) was the lowest, whereas that of EPO-IgG1 Fc (111.5 pM) was the highest (Fig. 3B). Consistent with a previous report [16], the *in vitro* bioactivity of darbepoetin alfa was lower (i.e., higher ED_50_, 84.4 pM) than r-EPO (52.9 pM) due to its high sialic acid content. Considering that one EPO-hyFc molecule contains two EPO molecules, ED_50_ of an individual EPO is 55.2 pM, which is comparable to that of r-EPO, indicating that there was no significant loss of bioactivity of EPO by hyFc-fusion. Also, colony formation assays were performed to determine the effect of EPO-hyFc on the proliferation of bone marrow erythroid progenitor cells by quantifying CFU-Es (late erythroid progenitors) and BFU-Es (early erythroid progenitors). One micromole of r-EPO and darbepoetin alfa treatment resulted in 131.8 and 105.3 CFU-E colonies, respectively (Fig. 3C). EPO-hyFc led to the highest number of CFU-E colonies (159.0), which is higher than those by EPO-IgG1 Fc treatment (123.5). Consistently, r-EPO and EPO-IgG1 Fc treatment induced comparable BFU-E colonies (15.0 and 14.7). In contrast, darbepoetin alfa and EPO-hyFc treatment generated the lowest (7.5) and the highest (22.7) numbers of BFU-E colonies, respectively. Taken together, these results suggest that EPO-hyFc is more effective in generating both CFU-E and BFU-E than EPO-IgG1 Fc, indicating the importance of a flexible hinge region.

**Figure 4:**
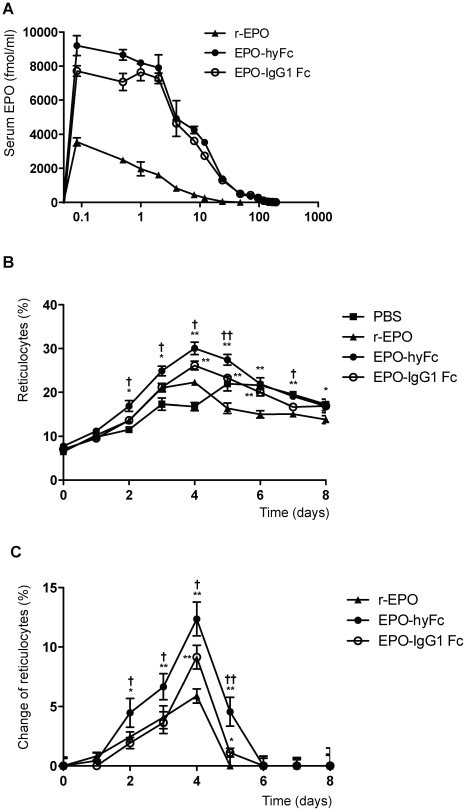
Pharmacokinetic and pharmacodynamic profiles of r-EPO, EPO-hyFc, and EPO-IgG1 Fc in rats. A single 400 pmol/kg dose of r-EPO (▴), EPO-hyFc (•), or EPO-IgG1 Fc (○) was administered in SD rats (n = 6/group), (A) serum EPO levels versus time were determined at the indicated time points by EPO ELISA. (B, C) Absolute reticulocyte counts (B) and changes in reticulocyte counts (C) in blood, expressed relative to the levels in buffer-treated rats after excluding the level determined prior to the administration, were evaluated by flow cytometry. Data, presented as means ± SEMs, were obtained from a single experiment. (*p<0.05, **p<0.01 compared with r-EPO; †p<0.05, ††p<0.01 compared with EPO-IgG1 Fc)

To compare the pharmacokinetic profiles of the two Fc-fused EPO proteins, we delivered a single 400-pmol/kg dose of r-EPO, EPO-hyFc, or EPO-IgG1 Fc into SD rats via the IV route. The serum half-life of r-EPO, used as a control, was 5.5 hours (Table 1, Fig. 4A), consistent with previous reports [14]. Notably, both EPO-IgG1 Fc and EPO-hyFc were cleared about five-times more slowly than r-EPO; moreover, the serum half-life of EPO-hyFc (29.1 hours) was longer than that of EPO-IgG1 Fc (24.7 hours). Additionally, the AUC_last_ (area under the concentration-time curve up to the last quantifiable concentration) values for EPO-IgG1 Fc and EPO-hyFc were about 9- and 10-fold larger than that for r-EPO, indicating that the fusion of either hyFc or IgG1 Fc increased the *in vivo* residence time of EPO.

**Table 1:** Pharmacokinetic parameters of r-EPO, EPO-hyFc and EPO-IgG1 Fc after IV administration into rats.

	Dose, pmol/kg	AUC_last_ a, hr×pmol/ml	C_0_ b, pmol/ml	t_1/2_ c, hr
r-EPO	400	13.6±0.4	3.6±0.2	5.5±0.2
EPO-hyFc	400	139.5±4.2	9.0±0.5	29.1±1.3
EPO-IgG1 Fc	400	127.6±3.3	7.7±0.3	24.7±0.4

a area under the concentration-time curve up to the last quantifiable concentration.

b initial observed serum concentration at 5 min.

c terminal serum half-life.

Concomitant with the pharmacokinetic study, the percentage of reticulocytes in blood, a marker of *in vivo* bioactivity of EPO, was measured daily by flow cytometry. As shown in Fig. 4B and 4C, the number of reticulocytes in EPO-IgG1 Fc-injected rats was increased at 4 and 5 dpi compared to r-EPO-injected rats, peaking at 4 dpi (relative increase of 9.35%). In contrast, EPO-hyFc significantly increased reticulocyte numbers from 2 to 5 dpi to an even greater extent (relative increase of 13.27%; peak at 4 dpi, p<0.01). PBS-treated group has a tendency to slightly increase reticulocytes, presumably due to the frequent bleeding for PK study, which is consistent with a previous report [30]. The AUC_last_ for the increase in reticulocytes generated by EPO-hyFc reached 29.8 d×%, which was approximately 2.3- and 1.7-fold higher than that generated by r-EPO (13.0 d×%) and EPO-IgG1 Fc (17.5 d×%), respectively. Taken together, our results indicate that the flexibility of the hyFc-hinge region may be mainly responsible for the better *in vivo* bioactivity of EPO-hyFc compared to EPO-IgG1 Fc.

### Differences in the pharmacokinetic and pharmacodynamic profiles of EPO-hyFc and darbepoetin alfa in rats

Because sialic acid content plays a role in the *in vivo* half-life of proteins, as shown in darbepoetin alfa, we separated purified EPO-hyFc into high and low sialic acid forms by Q sepharose chromatography. The high sialic acid form, EPO-hyFc(H), contained approximately 26 sialic acid residues/molecule, and the low sialic acid form, EPO-hyFc(L), contained about 16 sialic acids/molecule. An isoelectric focusing (IEF) analysis showed that the pI values for EPO-hyFc(H) and EPO-hyFc(L) were 4.4–5.0 and 5.1–6.6 pI, respectively (data not shown). As expected, EPO-hyFc(H) showed significantly enhanced *in vivo* bioactivity compared to EPO-hyFc(L) in BDF-1 mice after SC administration (Fig. S3). Consistent with this, we also found that EPO-hyFc(H) had a longer serum half-life and significantly higher C_0_ (initial observed serum concentration) than EPO-hyFc(L) (data not shown). These findings are also in agreement with a previous report that IgG2a Fc-EPO fusion molecules with higher sialic acid content isolated from BHK cells showed improved *in vivo* pharmacokinetic and pharmacodynamic profiles compared with those with lower sialic acid content isolated from NS/0 and 293 cells [31].

**Figure 5:**
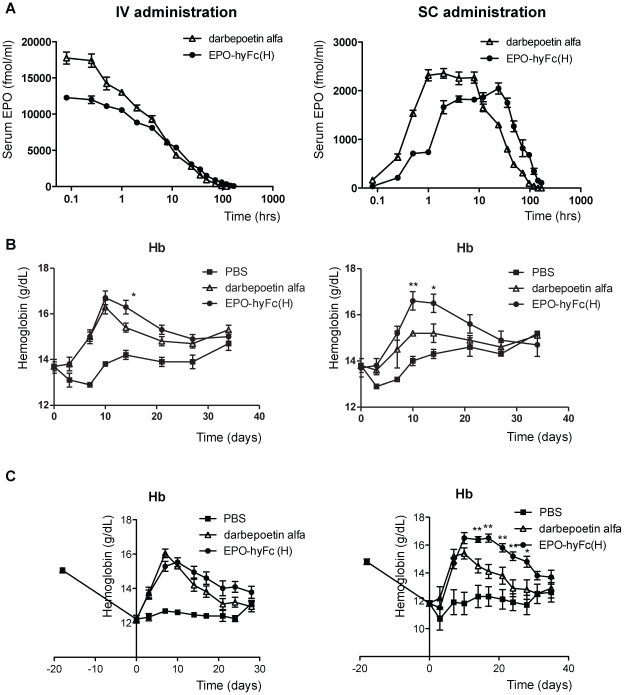
Pharmacokinetic and pharmacodynamic profiles of EPO-hyFc(H) and darbepoetin alfa in rats. (A) Serum EPO levels versus time after a single 800-pmol/kg dose of darbepoetin alfa (△) or EPO-hyFc(H) (•) administered IV or SC into SD rats (n = 10/group) were determined at the indicated time points (blood was collected from five rats each at alternate time points) by EPO ELISA. (B, C) The mean Hb concentrations versus time after the administration of darbepoetin alfa (△) or EPO-hyFc(H) (•) into normal SD rats (n = 5/group; 135-pmol/kg) (B) and cisplatin-induced anemic rats (n = 8 (IV) or 6 (SC) / group; 100-pmol/kg) (C) were evaluated at the indicated time points using an automated CBC counter. Changes in Hb concentration were expressed relative to the levels in buffer-treated rats after excluding the level determined prior to administration. Data, expressed as means ± SEMs, are representative of those obtained from two experiments. (*p<0.05, **p<0.01 compared with darbepoetin alfa)

Next, since darbepoetin alfa has been considered the gold standard for a long-acting, second-generation of EPO analogue, which has been extensively used in clinics, we performed a head-to-head comparison of the pharmacokinetic profiles of EPO-hyFc(H) and darbepoetin alfa (Table 2, Fig. 5A). The half-life of IV-administered EPO-hyFc(H) was significantly increased compared to darbepoetin alfa (25.3 vs. 13.8 hours, p<0.01); although the AUC_last_ for EPO-hyFc(H) trended higher (233.3 vs. 205.2 h·pmol/ml), this difference did not reach statistical significance. Unexpectedly, the C_0_ for EPO-hyFc(H) was less than that for darbepoetin alfa (11.2 vs. 18.6 pmol/ml, p<0.01), which may account for the lack of a significant difference in their AUC_last_ values despite the longer half-life of EPO-hyFc(H). EPO-hyFc(H) administered via the SC route exhibited a similar increase in serum half-life (31.6 vs. 15.6 hours, p<0.01), and also achieved a significantly higher AUC_last_ than darbepoetin alfa (138.1 vs. 92.2 h·pmol/ml, p<0.01). Although C_max_ (maximum observed serum concentration) of EPO-hyFc(H) was slightly lower than that of darbepoetin alfa (2.09 vs. 2.51 pmol/ml), the T_max_ (time of C_max_) of EPO-hyFc(H) was significantly delayed compared to darbepoetin alfa (30.8 vs. 17.6 hours, p<0.05). These results suggest that hyFc fusion mediates a longer duration of above-threshold EPO levels in blood. The relative bioavailability of EPO-hyFc(H), determined as the AUC_last_ after SC administration relative to the mean AUC_last_ after IV administration, was also significantly higher than that of darbepoetin alfa (59.2% vs. 44.9%, p<0.01), indicating that EPO-hyFc(H) is efficiently absorbed and utilized compared to darbepoetin alfa, especially following SC administration.

**Table 2:** PK parameters of darbepoetin alfa and EPO-hyFc(H) after IV and SC administration into rats.

	Route	Dose, pmol/kg	AUC_last_ a, hr×pmol/ml	C_max_ b, pmol/ml	C_0_ c, pmol/ml	T_max_ d, hr	t_1/2_ e, hr
Darbepoetin alfa	IV	800	205.2±3.9	NA	18.6±0.4	N/A	13.8±0.5
EPO-hyFc(H)	IV	800	233.3±2.2	NA	11.2±0.2	N/A	25.3±1.6
Darbepoetin alfa	SC	800	92.2±0.5	2.51±0.04	NA	17.6±1.8	15.6±1.0
EPO-hyFc(H)	SC	800	138.1±4.6	2.09±0.08	NA	30.8±3.5	31.6±1.6

NA ; not available.

a area under the concentration-time curve up to the last quantifiable concentration.

b maximum observed serum concentration.

c initial observed serum concentration at 5 min.

d time of maximum observed serum concentration.

e terminal serum half-life.

To compare the effects of EPO-hyFc(H) and darbepoetin alfa on *in vivo* erythropoiesis, we evaluated the profiles of serum Hb concentration versus time in rats after administering the same molar amounts of darbepoetin alfa or EPO-hyFc(H) via IV and SC routes (Fig. 5B). EPO-hyFc(H) administration led to a significantly higher Hb concentration than darbepoetin alfa at 14 dpi and the mean Hb concentration from 10 to 27 dpi following IV administration of EPO-hyFc(H) trended higher than that after IV administration of darbepoetin alfa, as did the AUC_last_ of the Hb increase (47.2 vs. 39.2 d·g/dL), although these differences were statistically insignificant. In contrast, SC administration of EPO-hyFc(H) induced a significantly greater Hb concentrations than did darbepoetin alfa at 10 and 14 dpi and significantly augmented the AUC_last_ of Hb increase compared to darbepoetin alfa (45.0 vs. 24.4 d·g/dL, p<0.01). Interestingly, EPO-hyFc(H) exhibited an *in vivo* bioactivity comparable to that of darbepoetin alfa even at a molar amount one-third that of darbepoetin alfa (Fig. S4). These results imply that, even considering that one EPO-hyFc molecule contains two EPO molecules, EPO-hyFc(H) is superior to darbepoetin alfa in terms of increased Hb levels, especially when administered via the SC route. To investigate *in vivo* erythropoiesis by darbepoetin alfa and EPO-hyFc(H) in cisplatin-induced anemic rats, rats were treated with 6 mg/kg cisplatin via intraperitoneal route, leading to decreased Hb concentration up to around 12 g/dL showing mild anemia. The mean Hb concentrations achieved by EPO-hyFc(H) administration via IV route appeared to be higher at 10 to 28 dpi than those by darbepoetin alfa, as did the AUC_last_ of the Hb increase (58.0 vs. 47.3 d·g/dL). More significantly, SC administration of EPO-hyFc(H) augmented Hb concentration from 14 to 28 dpi and AUC_last_ of Hb increase compared to darbepoetin alfa treatment (105.6 vs. 64.1 d·g/dL, p<0.01), which is consistent with the results obtained from normal rats, suggesting the possibility of delayed injection of EPO-hyFc(H) compared to darbepoetin alfa. Additionally, we confirmed that the increase in Hb levels and its duration by EPO-hyFc(H) after the induction of more severe anemia were comparable to that in mild anemic rats (Fig. S5). An increase in red blood cell counts and hematocrit by darbepoetin alfa and EPO-hyFc(H) in both normal and anemic rats showed a similar pattern with an increase in Hb concentration (Fig. S6). Taken together, these results indicate that EPO-hyFc(H) is highly effective in restoration from acute renal failure through active erythropoiesis.

**Figure 6:**
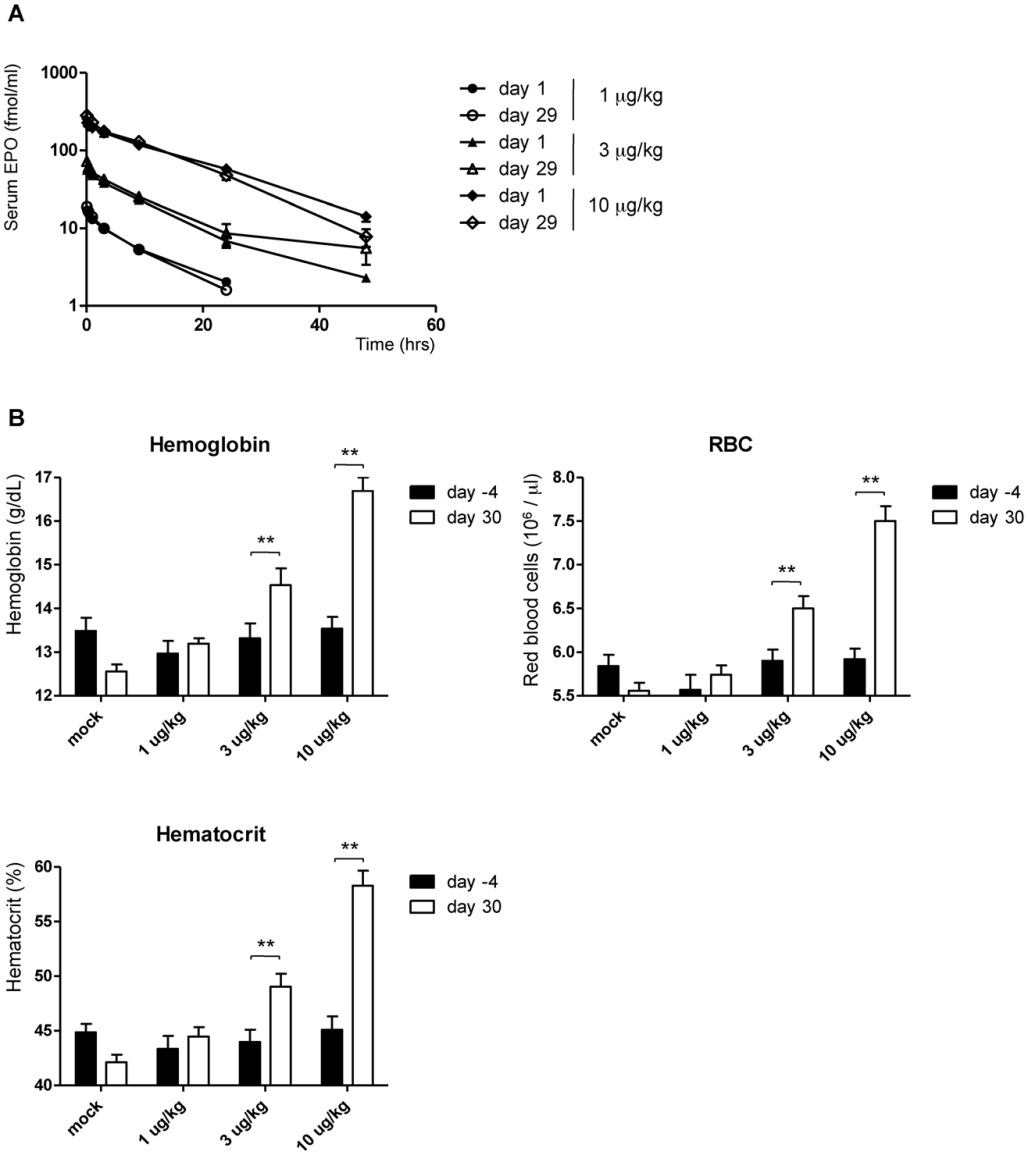
Pharmacokinetic and pharmacodynamic profiles of EPO-hyFc(H) in monkeys. (A) Serum EPO levels versus time after the first (day 1) and ninth (day 29) IV administration of 1, 3, and 10 µg/kg dose of EPO-hyFc(H) into cynomolgus monkeys (n = 10, buffer control and 10 µg/kg-treated groups; n = 6, 1 and 3 µg/kg-treated groups) were determined at the indicated time points by EPO ELISA. (B) The mean Hb concentrations, RBC counts and hematocrits before (day -4) or after nine times IV administration (day 30) of indicated dose of EPO-hyFc(H) into cynomolgus monkeys were evaluated using an ADVIA120 Hematology system. Data, expressed as means ± SEMs, are obtained from a single experiment. (*p<0.05, **p<0.01)

To examine the immunogenicity of EPO-hyFc(H) in cynomolgus monkeys, EPO-hyFc(H) was administered 9 times in three different doses (1, 3, and 10 µg/kg) via IV route. When EPO-hyFc(H)-specific antibody responses were determined by ELISA, there was no antibody induction specific to EPO-hyFc(H) in sera (Table 3), indicating that EPO-hyFc(H) would not be immunogenic. Additionally, there was no significant difference in the PK profiles of EPO-hyFc(H) administrated between at day 29 and at day 0, supporting the absence of EPO-hyFc-specific antibody induction (Fig. 6A). When we determined the effect of EPO-hyFc(H) on hematological responses, EPO-hyFc(H) treatment increased Hb concentrations, RBC counts and hematocrits in a dose-dependent manner (Fig. 6B).

**Table 3:** Induction of anti-EPO-hyFc antibody in cynomolgus monkeys.

	during treatment^a^			after treatment		
	day 1	day 15	day 29	Day 44	day 58	day 72
G1 (vehicle control)	0/10^b^	0/10	0/10	0/4	0/4	0/4
G2 (1 µg/kg EPO-hyFc(H))	0/6	0/6	0/6	N/A	N/A	N/A
G3 (3 µg/kg EPO-hyFc(H))	0/6	0/6	0/6	N/A	N/A	N/A
G4 (10 µg/kg EPO-hyFc(H))	0/10	0/10	0/10	0/4	0/4	0/4

NA ; not applicable.

a Nine times administration of EPO-hyFc(H) twice a week.

b Number of true positive animals/number of animals tested.

## Discussion

Human IgG1 Fc has been widely used in Fc-fusion strategies for developing long-acting antagonistic proteins, such as TNFR and CTLA-4, and provides a long half-life and improved efficacy [1]. However, its effector functions, including ADCC and CDC, would be disadvantageous when applied to agonistic proteins as this would lead to the death of target cells. A simple approach for overcoming this shortcoming is to directly mutate or delete sites related to ADCC and CDC [6]; but this comes at the cost of potential immunogenicity. Other methods include utilization of IgG4 Fc [7] or hybrid IgG2/IgG4 Fc [10], [11], but these could form monomeric molecules and/or hinder the bioactivity of Fc-fused molecules due to their less flexible hinge. More seriously, any hybrid Ig between IgG subclasses could not eliminate both ADCC and CDC completely. To circumvent these obstacles, we constructed a natural form of highly flexible hybrid Fc, hyFc, by replacing the hinge and the upper N-terminal CH2 domains of IgG4 Fc (which are essential for binding to all three human FcγRs) with the corresponding regions of IgD, a different class of Ig. Importantly, among human Igs, IgD shows the highest hinge-fold flexibility (77°) [32]. However, we previously found that the upper hinge region of IgD containing O-glycan moieties severely inhibited *in vivo* utilization after SC administration (unpublished data). We therefore used a partial hinge of IgD, which still contains considerable hinge-fold flexibility (53°) [32] compared to that of IgG1 (43°) and IgG2 (32°) [33], to construct hyFc.

Despite the low sequence homology (20.5%) between IgG4 Fc and IgD Fc, IgD exhibits a hydrophobicity profile similar to that of IgG4, especially in the upper CH2 region (Fig. S7). On the basis of a previous study that mutations do not disrupt Ig-like structure as long as the properties of amino acids in the hydrophobic/hydrophilic signature are conserved [34], we created a junction site between IgD and IgG4 in the hydrophobic region of the upper CH2 domain (Fig. S7) to maintain an intact hydrophobicity profile as well as to locate the junction site at inner space of hyFc. As expected, small X-ray scattering revealed that EPO-hyFc exhibited the “Y-shaped” structure like other Igs (Fig. 1B), and EPO-hyFc-specific antibody responses were not generated in monkeys intravenously injected 9 times (Table 3).

It has been previously reported that as the number of N-linked carbohydrates, especially terminal sialic acids, on EPO increased, receptor-binding activity decreased [35]. In addition, there was a positive correlation between receptor-binding activity and *in vitro* bioactivity and was the reason why darbepoetin alfa achieved less bioactivity than r-EPO in vitro. Here, EPO-hyFc showed similar *in vitro* bioactivity in the proliferation of F36E cells and erythroid progenitors compared to r-EPO, which might be due to the comparable sialic acid contents on EPO because any modification was not introduced on it. In contrast, EPO-IgG1 Fc exhibited less *in vitro* bioactivity than r-EPO and EPO-hyFc despite comparable sialic acid contents on EPO, reflecting the inhibitory effect of steric hindrance derived from IgG1 Fc.

Three major explanations have been proposed to account for the serum half-life of therapeutic proteins such as EPO. The first is receptor-mediated endocytosis and subsequent intracellular degradation; EPO is degraded only by EPOR-expressing cells, which include erythroid progenitor cells and non-hematopoietic cells such as neurons, glia, myoblasts, and endothelial cells [36]. To date, the identity of cells that are mainly involved in EPOR-mediated clearance has not yet been clearly established. The second mechanism is rapid elimination of desialylated EPO via an asialoglycoprotein receptor (ASGR) expressed on hepatocytes. Consistent with this possibility, there is a positive correlation between the number of sialic acids on EPO molecules and serum half-life [35]. Last is hydrodynamic size-dependent renal clearance: as the molecular size increases, the clearance in kidney is decreased [4]. In contrast to darbepoetin alfa, which is able to sustain a relatively long *in vivo* half-life due to its greater sialic acid content (22 vs. 14 residues) and a larger molecular size (37.4 vs. 30 kDa) compared to EPO [16], additional mechanisms may account for the longer serum half-life of EPO-hyFc. Although the larger hydrodynamic size of EPO-hyFc (130 kDa) compared to darbepoetin alfa (37.4 kDa) may contribute, FcRn-mediated recycling may play a major role in the longer serum half-life of EPO-hyFc, as evidenced by the significantly shorter serum half-life of EPO-hyFc in FcRn-deficient mice than in wild-type mice (data not shown). Unexpectedly, we found that the initial serum levels of EPO-hyFc(H) were significantly lower than those of darbepoetin alfa after IV injection, even though the larger hydrodynamic size of EPO-hyFc is predicted to inhibit renal clearance and extravasation. One explanation for this phenomenon is that EPO-hyFc may be internalized via FcRn into endothelial cells and hematopoietic cells soon after IV injection and is thus sequestered from blood. Alternatively, EPO-hyFc(H) may be cleared via ASGRs in the liver owing to the fact that its moieties are more asialylated than those of darbepoetin alfa (8∼11 vs. 0∼4 residues [37]). Previous studies showing that ASGR-mediated endocytosis and clearance occurs in a matter of minutes [38] suggest that the latter explanation may be more plausible. Additional support for this interpretation is provided by our observation that the C_0_ of EPO-hyFc(L) is significantly lower than that of EPO-hyFc(H).

It was reported that EPO-mimicking human antibody interacts with different sites on EPOR compared to an EPO molecule, but mediate the proliferation of EPO-responsive F36E cells and increase hematocrits after administration [27]. In terms of pharmacokinetics, EPO-mimicking antibody has a serum half-life of approximately 6 days, which is a significantly longer than darbepoetin alfa and EPO-hyFc(H). In contrast, EPO-mimicking antibody showed 10-fold lower *in vitro* bioactivity than r-EPO, while EPO-hyFc exhibited better activity than r-EPO on an equivalent molar basis. In addition, although EPO-mimicking antibody achieved better *in vivo* bioactivity than darbepoetin alfa, leading to less frequent injections, approximately 80-fold more EPO-mimicking antibody on a mass concentration was used. In contrast, an equivalent mole of EPO-hyFc(H), which is about 3-fold more on a mass concentration, induced better in vivo bioactivity than darbepoetin alfa in rats, suggesting that EPO-hyFc has an advantage in increasing bioactivity compared to EPO-mimicking antibody. However, it is to early to conclude relative effect in bioactivity due to the differences in experimental scheme. Therefore, it would be interesting to perform a head-to-head comparison between EPO-mimicking antibody and EPO-hyFc.

It has been reported that EPO mediates the dimerization of EPOR via asymmetric binding, followed by the activation of a signaling pathway [39]. Two adjacent EPO molecules by short linkers (3 to 7 aa) led to a moderate decrease in *in vitro* bioactivity of EPO-EPO fusion molecules compared to EPO monomers [40]. In contrast, the usage of 17 aa linker for EPO-EPO induced 6-fold higher bioactivity than the monomer, although the exact mechanisms have not yet been elucidated [41]. Similarly, we found that one EPO-hyFc molecule having a 30 aa flexible hinge region showed about 2-fold higher *in vitro* bioactivity than r-EPO, suggesting no inhibitory effect of hyFc-fusion in the dimerization of EPOR by each EPO molecule.

The bioavailability of r-EPO and darbepoetin alfa following SC administration increased in a dose-dependent manner [42]. Therefore, head-to-head comparisons of the bioavailability using molar-equivalent amounts of darbepoetin alfa and EPO-hyFc(H) were important in providing information regarding the suitability of SC administration. Interestingly, EPO-hyFc(H) exhibited significantly higher bioavailability than darbepoetin alfa (59.2% vs. 44.9%, p<0.01) after SC administration in rats. Previous reports have suggested that there is a negative correlation between hydrodynamic size and bioavailability [43], [44]. In addition, it has been reported that a high positive charge causes nonspecific adhesion of proteins to the extracellular matrix and inhibits their transport into the blood [45]. Despite the larger hydrodynamic size and higher pI values of EPO-hyFc(H) than darbepoetin alfa, which would be presumed to impede absorption after SC administration compared to darbepoetin alfa, the bioavailability of EPO-hyFc(H) was enhanced, possibly reflecting FcRn-mediated internalization. A previous report demonstrated that the bioavailability of monoclonal IgG1 antibody was significantly decreased in FcRn-deficient mice compared to that in wild-type mice (28.3% vs. 82.5% in FcRn-deficient vs. wild-type mice, respectively) [46]. In addition, it has been shown that FcRn is mainly expressed in the endothelial cells of small arterioles and capillaries, and that FcRn-binding proteins are predominantly localized in skin and muscle and, to a lesser extent, in liver and adipose tissue [47]. It is not yet known whether the effect of FcRn on SC bioavailability is mainly associated with FcRn-mediated protection from degradation or FcRn-mediated transport from the interstitial fluid to the blood through the vascular endothelium. However, the former mechanism is more conceivable because EPO-hyFc(H) showed a delayed T_max_ compared to darbepoetin alfa.

## Supporting Information

Figure S1 **Characterization of EPO-hyFc by Western blotting.** Western blot analyses of r-EPO, darbepoetin alfa, EPO-hyFc, and EPO-IgG1 Fc using anti-EPO antibody and anti-human IgG antibodies.
(TIFF)

Figure S2 **Histological findings in bone marrow after the treatment of EPO-hyFc(H) into monkeys.** Representative histologies from the sternum of male cynomolgus monkeys treated with 1, 3, 10 mg/kg of EPO-hyFc(H) or vehicle control in a 31-day-toxicity study were shown. Preserved tissues in neutral buffered 10% formalin were embedded in paraffin, sectioned, stained with hematoxylin and eosin (H&E), and examined microscopically (lens magnification 80×; Eclipse 801i, Nikkon, Japan).
(TIFF)

Figure S3 **Pharmacodynamic profiles of EPO-hyFc in BDF-1 mice as a function of sialic acid content.** Male BDF-1 mice (n = 8/group) were injected SC with 50, 100, or 200 ng/ml dose of EPO-hyFc(H) (white column) or EPO-hyFc(L) (black column), and reticulocytes were counted using flow cytometry. Data, presented as means ± SEMs, are representative of two independent experiments. (*p<0.05, **p<0.01).
(TIFF)

Figure S4 **Pharmacodynamic profiles of one-third mole of EPO-hyFc(H) compared to darbepoetin alfa in rats.** Changes in Hb concentrations versus time after the administration of 135-pmol/kg of darbepoetin alfa (△) or 45 pmol/kg of EPO-hyFc(H) (•) into SD rats (n = 5/group) were evaluated at indicated time points by automated CBC counter. Changes in Hb concentrations were expressed relative to the levels in buffer-treated rats after excluding the level determined prior to administration. Data, presented as means ± SEMs, are representative of those obtained from two experiments.
(TIFF)

Figure S5 **Pharmacodynamic profiles of EPO-hyFc(H) and darbepoetin alfa in severe anemic rats.** The mean Hb concentrations versus time after the IV administration of 100 pmol/kg of darbepoetin alfa (△) or EPO-hyFc(H) (•) into severe anemic rats induced by 7 mg/kg cisplatin were evaluated at the indicated time points using an automated CBC counter. Data, expressed as means ± SEMs, are obtained from a single experiment. (*p<0.05 compared with darbepoetin alfa).
(TIFF)

Figure S6 **Change of RBC counts and hematocrits after treatment with EPO-hyFc(H) and darbepoetin alfa in normal and cisplatin-induced anemic rats.** (A, B) The mean RBC counts and hematocrits versus time after the administration of darbepoetin alfa (△) or EPO-hyFc(H) (•) into normal SD rats (n = 5/group; 135-pmol/kg) (A) and cisplatin-induced anemic rats (n = 8 (IV) or 6 (SC) /group; 100-pmol/kg) (B) were evaluated at the indicated time points using an automated CBC counter, respectively. Data, expressed as means ± SEMs, are representative of those obtained from two experiments. (*p<0.05, **p<0.01 compared with darbepoetin alfa).
(TIFF)

Figure S7 **Grand average hydrophobicity of IgD, IgG4, and hyFc.** The hydrophobicities of IgD, IgG4, and hyFc were determined using ProtScale tool (http://www.exapsy.org/tools). The red box denotes the partial region of IgD and IgG4 consisting of hyFc, and the arrow indicates the junction site between IgD and IgG4 in hyFc. A higher score indicates a greater degree of hydrophobicity. Black arrow indicates the junction site between IgD and IgG4.
(TIFF)
