# Supplementary figures and images for: Natural Form of Noncytolytic Flexible Human Fc as a Long-Acting Carrier of Agonistic Ligand, Erythropoietin
Source: PLoS One. 2011 Sep 16;6(9):e24574. doi: 10.1371/journal.pone.0024574 (PMC3174958; doi:10.1371/journal.pone.0024574)

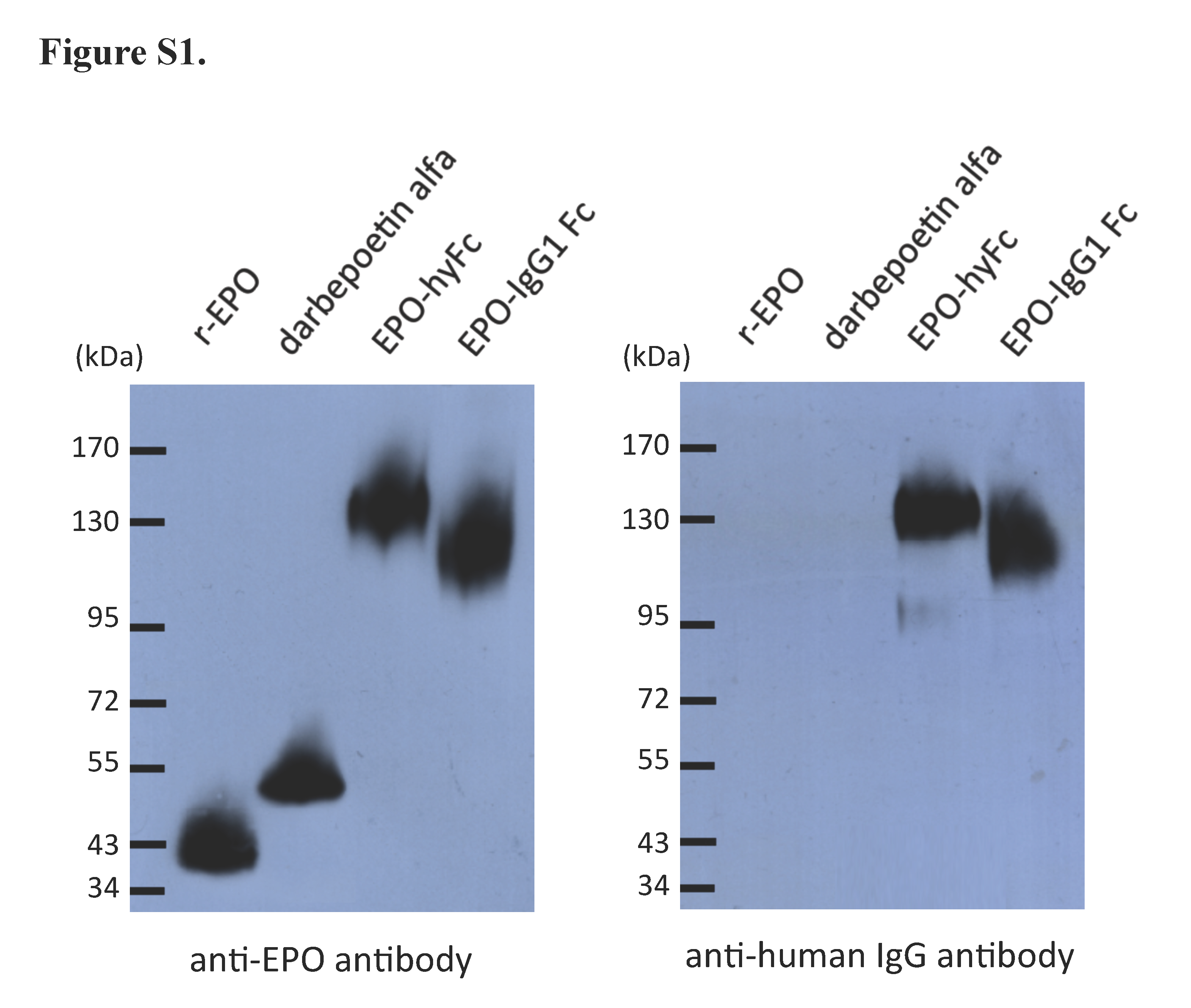

Supplement: Figure S1 — Characterization of EPO-hyFc by Western blotting. Western blot analyses of r-EPO, darbepoetin alfa, EPO-hyFc, and EPO-IgG1 Fc using anti-EPO antibody and anti-human IgG antibodies. (TIFF) [file pone.0024574.s001.tif]

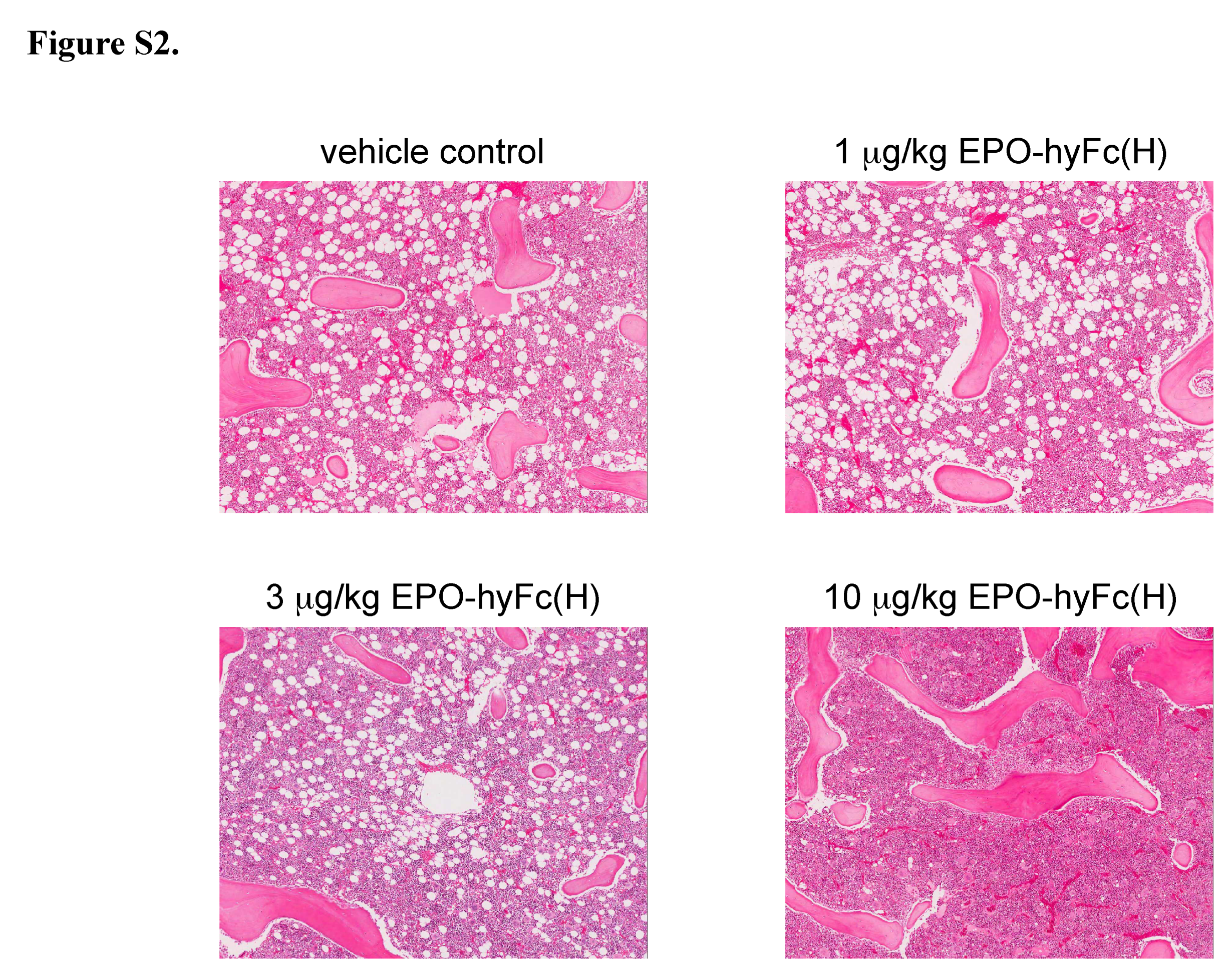

Supplement: Figure S2 — Histological findings in bone marrow after the treatment of EPO-hyFc(H) into monkeys. Representative histologies from the sternum of male cynomolgus monkeys treated with 1, 3, 10 mg/kg of EPO-hyFc(H) or vehicle control in a 31-day-toxicity study were shown. Preserved tissues in neutral buffered 10% formalin were embedded in paraffin, sectioned, stained with hematoxylin and eosin (H&E), and examined microscopically (lens magnification 80×; Eclipse 801i, Nikkon, Japan). (TIFF) [file pone.0024574.s002.tif]

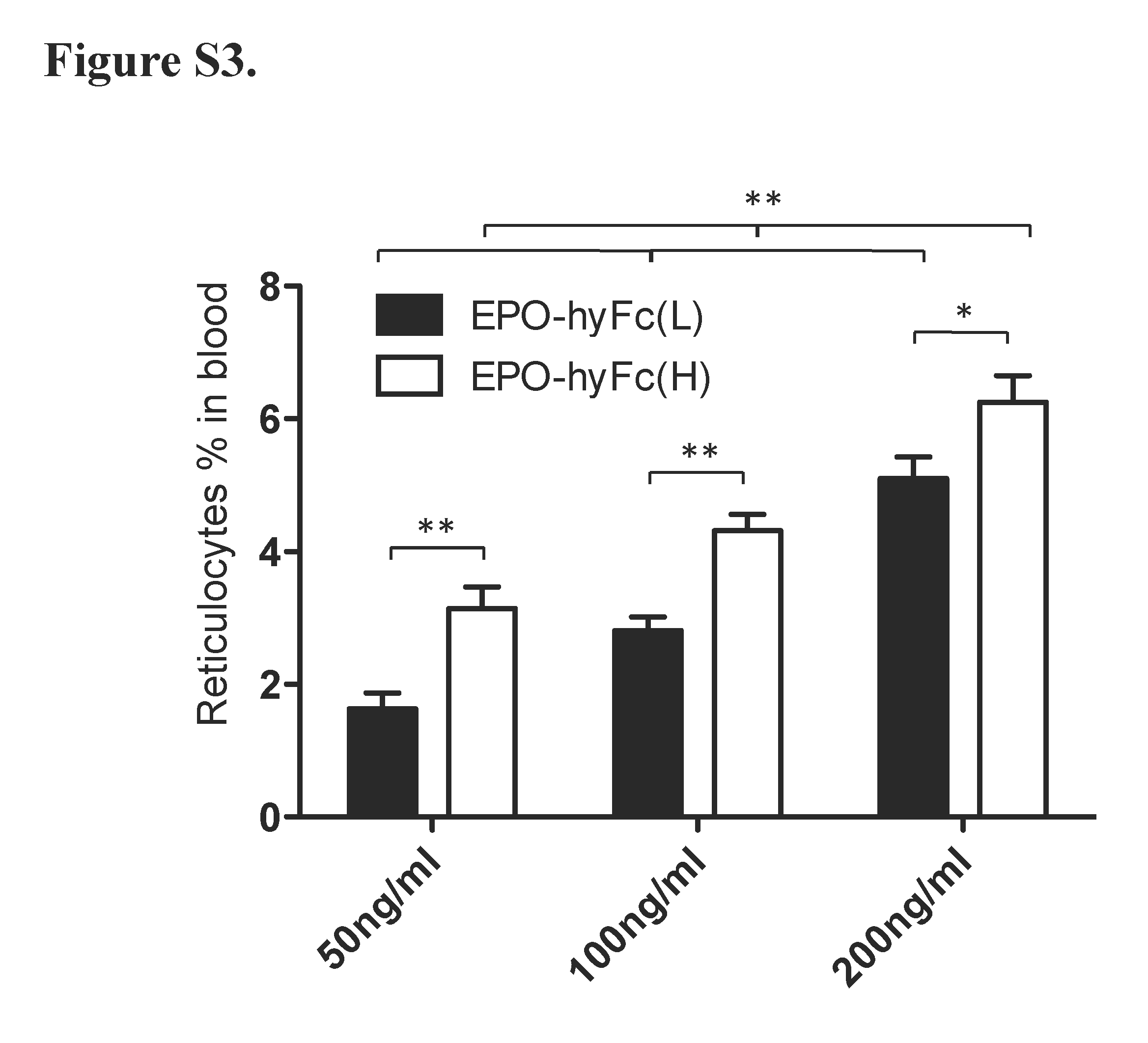

Supplement: Figure S3 — Pharmacodynamic profiles of EPO-hyFc in BDF-1 mice as a function of sialic acid content. Male BDF-1 mice (n = 8/group) were injected SC with 50, 100, or 200 ng/ml dose of EPO-hyFc(H) (white column) or EPO-hyFc(L) (black column), and reticulocytes were counted using flow cytometry. Data, presented as means ± SEMs, are representative of two independent experiments. (*p<0.05, **p<0.01). (TIFF) [file pone.0024574.s003.tif]

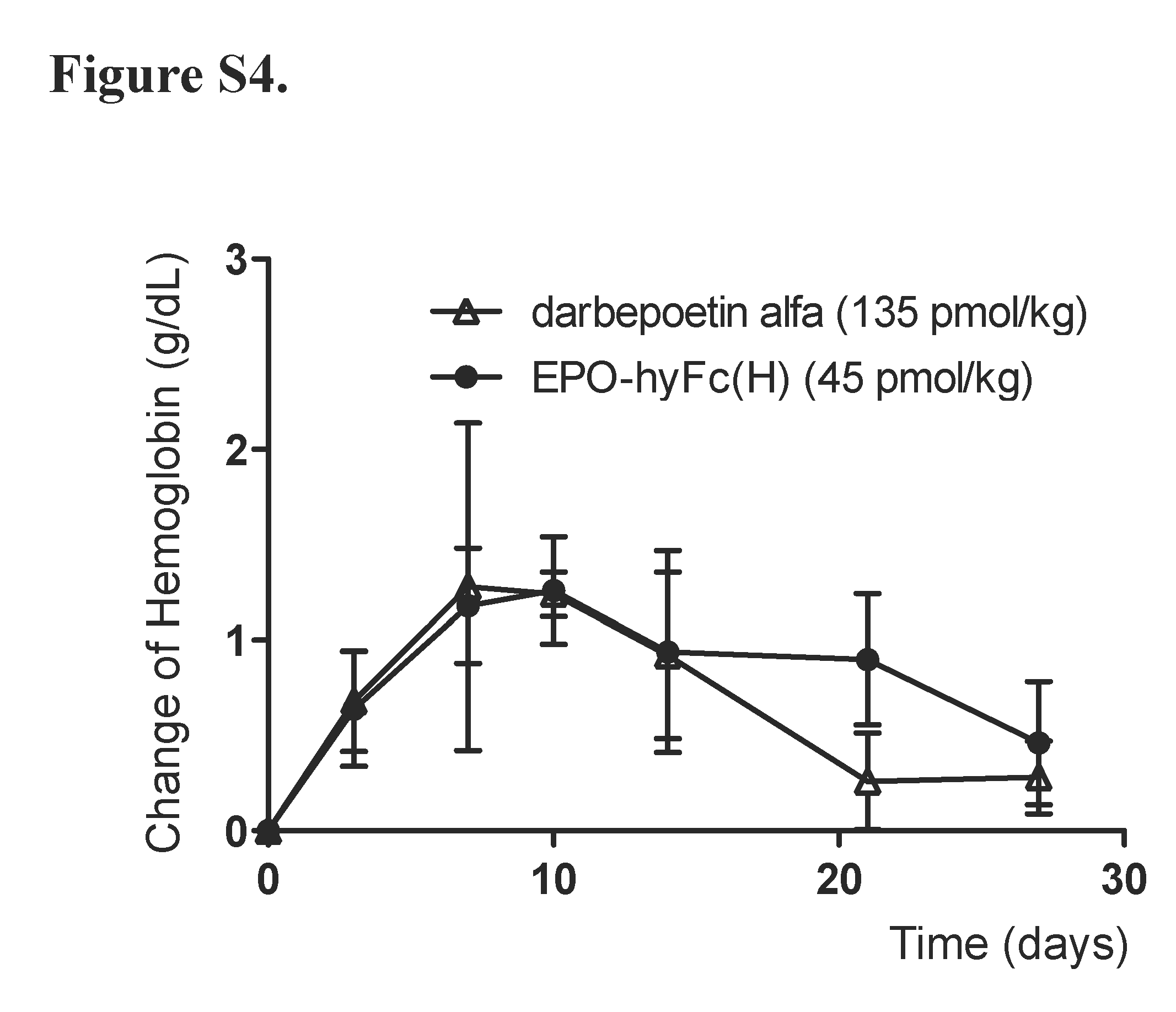

Supplement: Figure S4 — Pharmacodynamic profiles of one-third mole of EPO-hyFc(H) compared to darbepoetin alfa in rats. Changes in Hb concentrations versus time after the administration of 135-pmol/kg of darbepoetin alfa (△) or 45 pmol/kg of EPO-hyFc(H) (•) into SD rats (n = 5/group) were evaluated at indicated time points by automated CBC counter. Changes in Hb concentrations were expressed relative to the levels in buffer-treated rats after excluding the level determined prior to administration. Data, presented as means ± SEMs, are representative of those obtained from two experiments. (TIFF) [file pone.0024574.s004.tif]

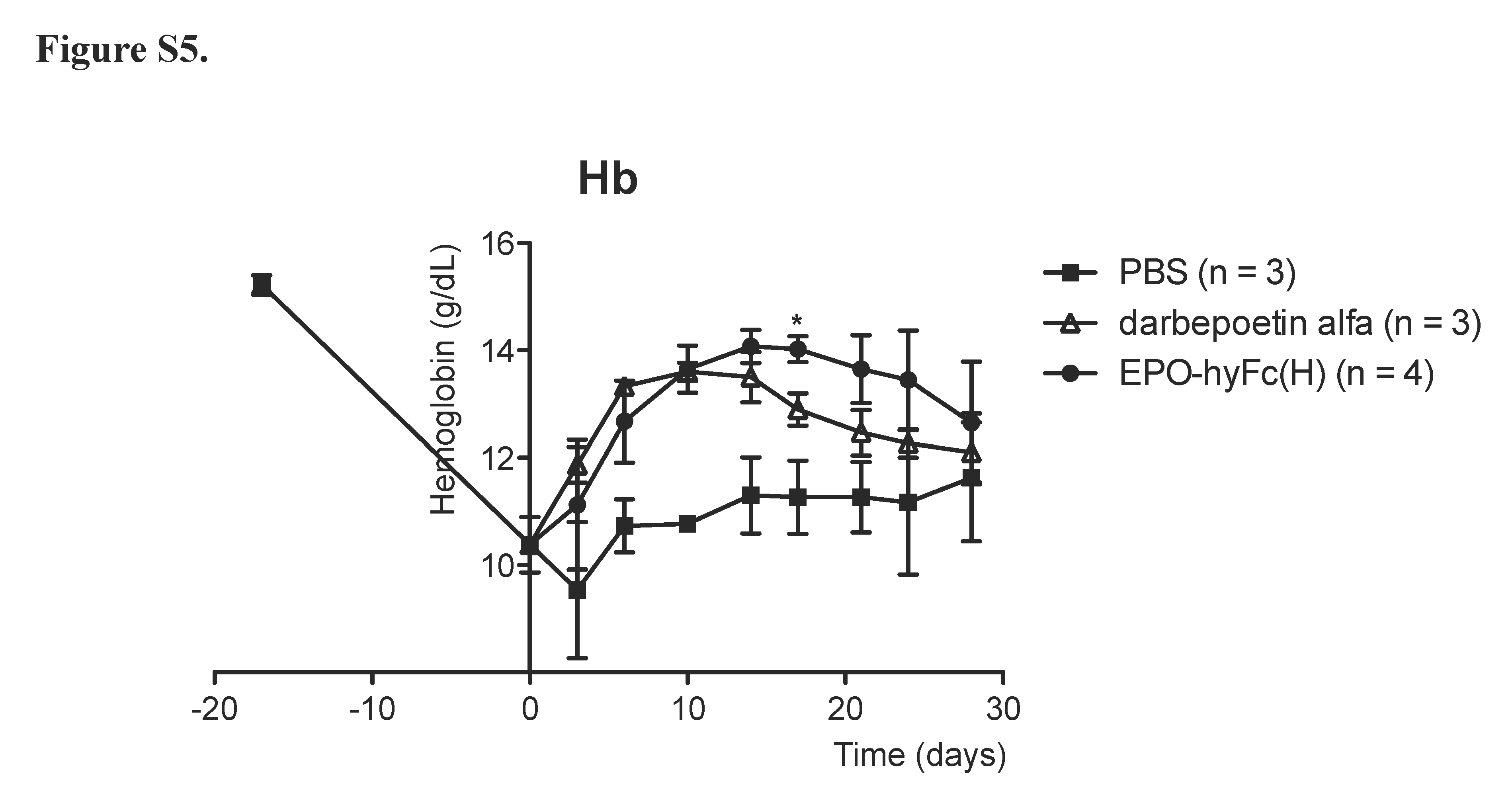

Supplement: Figure S5 — Pharmacodynamic profiles of EPO-hyFc(H) and darbepoetin alfa in severe anemic rats. The mean Hb concentrations versus time after the IV administration of 100 pmol/kg of darbepoetin alfa (△) or EPO-hyFc(H) (•) into severe anemic rats induced by 7 mg/kg cisplatin were evaluated at the indicated time points using an automated CBC counter. Data, expressed as means ± SEMs, are obtained from a single experiment. (*p<0.05 compared with darbepoetin alfa). (TIFF) [file pone.0024574.s005.tif]

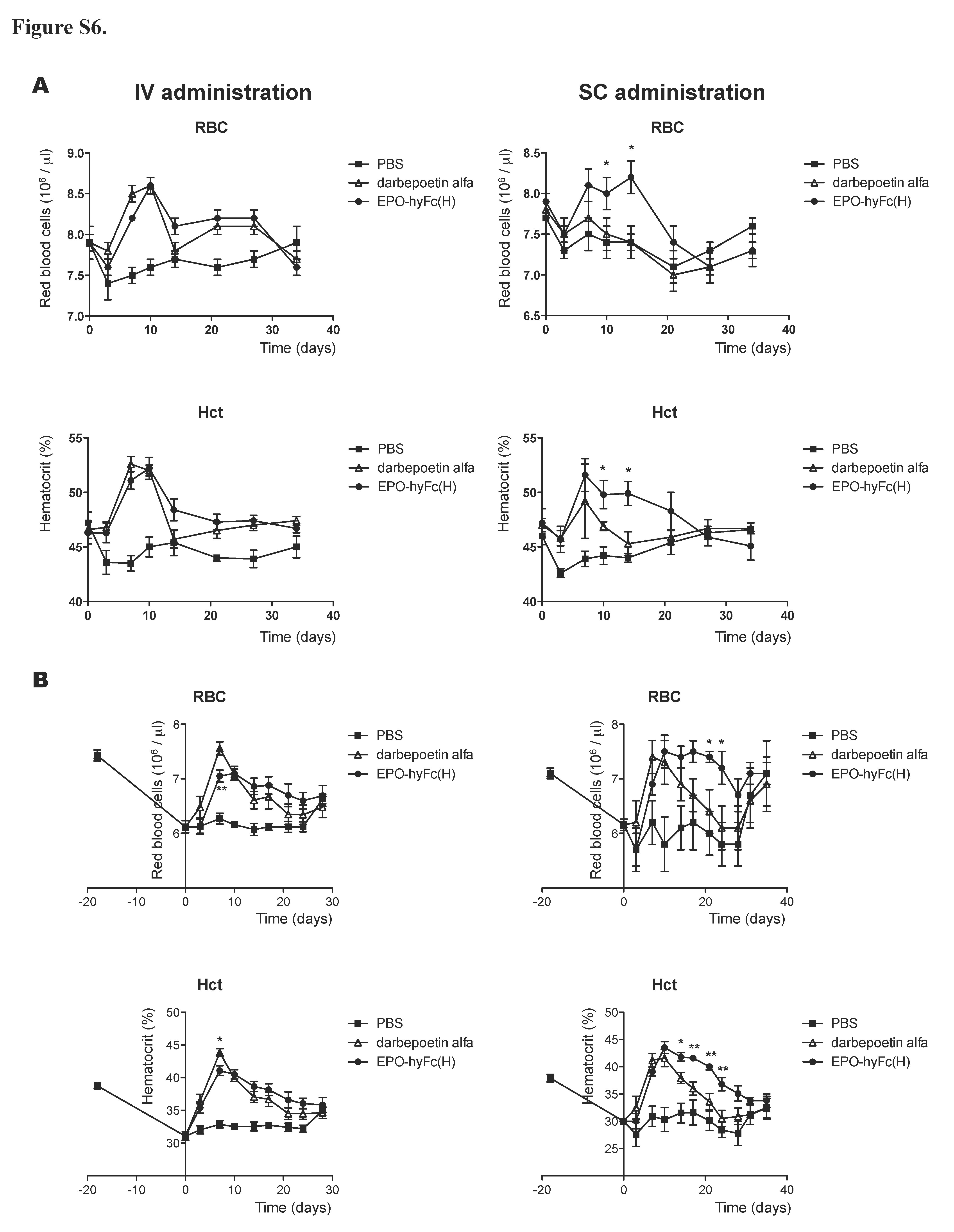

Supplement: Figure S6 — Change of RBC counts and hematocrits after treatment with EPO-hyFc(H) and darbepoetin alfa in normal and cisplatin-induced anemic rats. (A, B) The mean RBC counts and hematocrits versus time after the administration of darbepoetin alfa (△) or EPO-hyFc(H) (•) into normal SD rats (n = 5/group; 135-pmol/kg) (A) and cisplatin-induced anemic rats (n = 8 (IV) or 6 (SC) /group; 100-pmol/kg) (B) were evaluated at the indicated time points using an automated CBC counter, respectively. Data, expressed as means ± SEMs, are representative of those obtained from two experiments. (*p<0.05, **p<0.01 compared with darbepoetin alfa). (TIFF) [file pone.0024574.s006.tif]

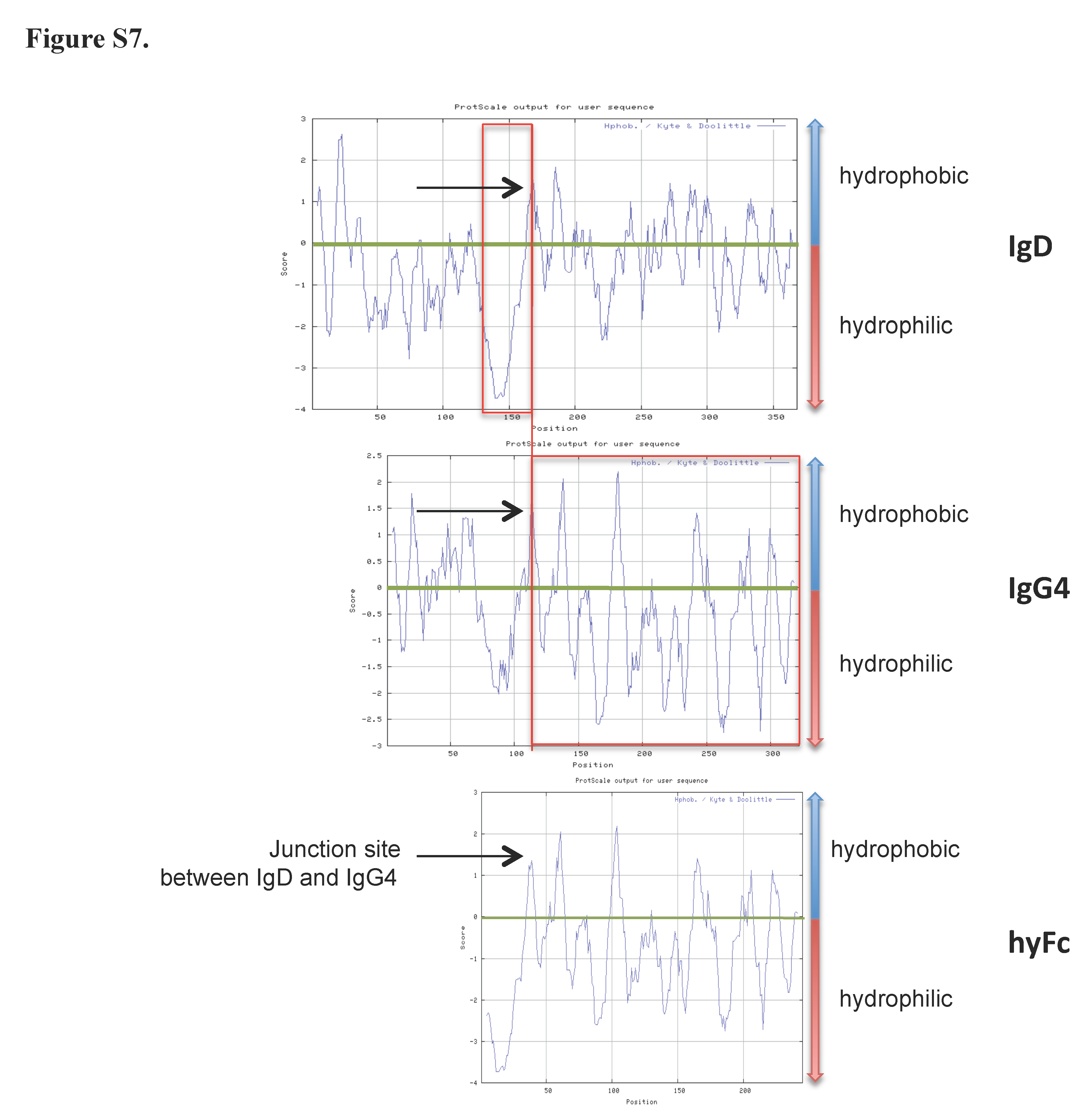

Supplement: Figure S7 — Grand average hydrophobicity of IgD, IgG4, and hyFc. The hydrophobicities of IgD, IgG4, and hyFc were determined using ProtScale tool (http://www.exapsy.org/tools). The red box denotes the partial region of IgD and IgG4 consisting of hyFc, and the arrow indicates the junction site between IgD and IgG4 in hyFc. A higher score indicates a greater degree of hydrophobicity. Black arrow indicates the junction site between IgD and IgG4. (TIFF) [file pone.0024574.s007.tif]
